# A Delphi consensus panel about clinical management of early-stage EGFR-mutated non-small cell lung cancer (NSCLC) in Spain: a Delphi consensus panel study

**DOI:** 10.1007/s12094-022-02941-5

**Published:** 2022-09-27

**Authors:** Dolores Isla, Enriqueta Felip, Pilar Garrido, Amelia Insa, Margarita Majem, Jordi Remon, Jose M. Trigo, Javier de Castro

**Affiliations:** 1 Hospital Universitario Lozano Blesa, IIS Aragón, Saragossa, Spain; 2 Hospital Vall d’Hebron, Barcelona, Spain; 3 Hospital Universitario Ramón y Cajal, Madrid, Spain; 4 Hospital Clínico Universitario de Valencia, Valencia, Spain; 5 Hospital de la Santa Creu i Sant Pau, Barcelona, Spain; 6 CIOCC HM Nou Delfos, Barcelona, Spain; 7 HC Marbella-Hospital International, Málaga, Spain; 8 Hospital Universitario La Paz-IDIPAZ, Madrid, Spain

**Keywords:** Adjuvant, Consensus, Delphi method, Non-small cell lung cancer (NSCLC), osimertinib, *EGFR*-mutated

## Abstract

**Purpose:**

This Delphi panel study assessed the level of consensus between medical oncologists on the clinical management of patients with early-stage *EGFR*-mutated non-small cell lung cancer (NSCLC).

**Methods:**

A modified two-round Delphi approach was used. A scientific committee comprised of medical oncologists developed an online questionnaire. Delphi panel experts rated their level of agreement with each questionnaire statement on a 9-point Likert scale. The questionnaire included 36 statements from 3 domains (clinical management of early-stage NSCLC: 15 statements; role of adjuvant therapy in early-stage NSCLC: 9 statements; and role of adjuvant therapy in early-stage NSCLC with sensitizing *EGFR* mutation: 12 statements).

**Results:**

In round 1, consensus was reached for 24/36 statements (66.7%). Nine statements that did not achieve consensus after the first round were evaluated in round 2, and none of them reached consensus. Overall, 84.4% of the panelists agreed that *EGFR* mutation testing should be done after surgery. Consensus was not achieved on whether the implementation of *EGFR* mutation testing in resected early-stage NSCLC could limit the use of adjuvant osimertinib. The panelists recognized the rationale for the use of osimertinib in the adjuvant scenario (88%) and 72% agreed that it may change the treatment paradigm in stage IB–IIIA *EGFR*-mutated NSCLC. Consensus was not reached on the inconvenience of prolonged duration of osimertinib.

**Conclusions:**

This Delphi study provides valuable insights into relevant questions in the management of early-stage *EGFR*-mutated NSCLC. However, specific issues remain unresolved. The expert consensus emphasizes the role of adjuvant treatment with osimertinib in this scenario.

**Supplementary Information:**

The online version contains supplementary material available at 10.1007/s12094-022-02941-5.

## Introduction

Early-stage non-small cell lung cancer (NSCLC) represents approximately 25% of all new lung cancer diagnoses [1], and for fit patients, surgery remains the cornerstone treatment. Perioperative platinum-based chemotherapy improves the 5-year overall survival (OS) by 5% [2, 3], regardless of timing of chemotherapy administration (adjuvant versus neoadjuvant) [4], and it is recommended in patients with stage II–IIIA NSCLC [1]. However, even for those patients treated with adjuvant chemotherapy, the 5-year OS remains 65% [3], and local and distant recurrence rates are high [5]. Despite several strategies tested over time in this setting, none has changed the standard of care or improved the patients’ outcomes. In contrast, the recent introduction of targeted therapies in oncogene-addicted tumors and immune checkpoint inhibitors in early-stage NSCLC, either in the neoadjuvant setting in combination with chemotherapy or in the adjuvant setting in PD-L1-positive tumors after chemotherapy, have shifted the treatment paradigm and outcome in this population [6–8].

Since 2020, based on the ADAURA trial, both, the European Medicines Agency (EMA) and the Food and Drug Administration (FDA) approved adjuvant treatment with osimertinib for 3 years, a third-generation epidermal growth factor receptor (*EGFR*) tyrosine kinase inhibitor (TKI) for patients with completely resected stage IB–IIIA NSCLC harboring a sensitizing *EGFR* mutation. *EGFR* mutations are the most prevalent drivers of NSCLC oncogenicity, occurring in up to 15–20% of patients with adenocarcinoma [9], varying significantly across different geographic locations [10–13]. This approval was based on a significant improvement in disease-free survival (DFS) in patients treated with osimertinib compared with placebo [Hazard ratio (HR) 0.20, 99.12% CI 0.14–0.30; *P* < 0.001] [14], and a reduced risk of developing central nervous system (CNS) metastases (1% vs. 10%, respectively) [15]. Of note, the DFS benefit with osimertinib was observed regardless of tumor stage or the use of adjuvant chemotherapy [14]. Additionally, adjuvant osimertinib did not negatively impact health-related quality of life (HRQoL) compared with placebo [16]. Although findings from the ADAURA trial are clinically meaningful, and osimertinib is an accepted potential strategy among some physicians, [17] this trial has also raised several challenging clinical questions that remain unresolved. First, the overall survival benefit with adjuvant osimertinib remains unknown since OS data are still immature. Second, in patients receiving targeted therapies, DFS may not be a valid surrogate endpoint for OS. Additionally, the use of osimertinib in early stage may negatively impact in subsequent treatment lines at the time of onset of metastatic disease. Finally, three years of adjuvant treatment may induce unavoidable financial toxicity [18].

Due to controversies surrounding the use of adjuvant osimertinib in patients with early-stage EGFR-mutated NSCLC, we performed a Delphi survey to explore expert opinions regarding the current treatment approach in this setting. Indeed, we aimed to assess the potential advantages of the use of adjuvant osimertinib, as well as the potential limitations regarding its incorporation in the adjuvant setting.

## Methods

### Study design

The EARLY project was a national multicenter two-round Delphi panel study to seek expert-based opinions regarding clinical management of early-stage NSCLC (7th TNM edition). We aimed to develop a consensus on its management, goals, and impact of adjuvant therapy, with a particular focus on the role of osimertinib in the adjuvant scenario.

The Delphi method is a widely accepted scientific, structured, and systematic technique to gain consensus when there is limited or conflicting available evidence. The Delphi method consists of an iterative process comprising multiple rounds of controlled, individual, and anonymous feedback from a group of experts through structured questionnaires [19, 20]. We applied a Delphi study involving two structured rounds of questions for obtaining a consensus of opinions from a group of geographically dispersed experts (Delphi expert panel) using an online questionnaire through a web platform. This study followed the RAND/UCLA Delphi panel method [21] for gathering consensus among experts.

### Delphi experts

The scientific committee was comprised of eight medical oncologists with recognized expertise in lung cancer management and research. The scientific committee was involved in the following steps of this Delphi project: (1) extensive literature review for preparing/designing the Delphi questionnaire; (2) generation of questionnaire domains and statement; (3) definition of the consensus level and Delphi methodology; (4) selection of the Delphi expert panel; (5) interpretation and discussion of the results of the Delphi questionnaire after each round; and (6) development of final consensus document.

The Delphi expert panel consisted of medical oncologists specialized on lung cancer and outstanding for their clinical research from hospitals distributed throughout Spain. In order for the experts to be selected for the Delphi panel, they needed to provide care for a large number of lung cancer patients at tertiary-level hospitals, with highly specialized staff and technical equipment, where EGFR mutation testing is feasible and routinely performed. On April 26, 2021, a total of 32 experts were invited to take part in the project. Of these, 31 (96.8%) experts completed the second round. Experts who agreed to participate received an electronic link providing personalized access to the online platform.

### Delphi questionnaire domain/item generation

The scientific committee conducted an extensive literature search regarding relevant topics on the clinical management of early-stage NSCLC, focusing on adjuvant therapy. The final Delphi questionnaire included 36 statements grouped in 3 major domains:

1. Clinical management of early-stage NSCLC: 15 statements
2. Role of adjuvant therapy in early-stage NSCLC: 9 statements
3. Role of adjuvant therapy in early-stage NSCLC with sensitizing *EGFR* mutation: 12 statements.

### Two-round Delphi process

The Delphi process involved two rounds of questioning of panel experts using an online platform. In round 1, Delphi expert panel was asked to rate their level of agreement with each questionnaire statement on a 9-point Likert scale from 1 (completely disagree) to 9 (completely agree). The expert panel individually and anonymously provided feedback to each questionnaire statement based on routine oncology practice and current clinical evidence.

The first-round results were discussed by the scientific committee in a meeting in June 2021. For each questionnaire statement, consensus was considered to have been achieved based on the agreement of at least 66.6% of the expert panel and the acceptance of the scientific committee. Those statements that did not achieve consensus were removed or modified.

In round 2, the updated questionnaire, which included statements that did not reach consensus in the first round, was redistributed for the re-evaluation of these statements. All statements reaching consensus in round 1 were removed. Panelists were asked to rate again statements that had not reached consensus using the same voting method described for round 1. After analyzing the second-round data, statements that lacked consensus were discussed by the scientific committee in a meeting (round 3).

### Statistical analysis

The level of agreement of the Delphi panelists with each questionnaire item was categorized according to the scores on the 9-point scale. Statements scored in the 1–3 range were classified as rejected, those in the 4–6 range as undetermined/uncertain, and those in the 7–9 range as accepted. The percentage of panel experts rating in the range of 1–3, 4–6, and 7–9, respectively, was calculated to assess the degree of consensus for each item. Consensus was achieved when at least 66.6% of panelists reached agreement (ratings of 7–9) or disagreement (ratings of 1–3).

All the statistical analyses were performed using the Statistical Package for the Social Sciences (SPSS) version 18.0 (SPSS Inc., Chicago, IL, USA).

## Results

In round 1 (May 2021), consensus was reached for 24/36 statements (66.7%). Nine statements that did not achieve consensus after round 1 were evaluated in round 2 (June–August 2021). None of the statements evaluated in round 2 reached consensus.

### Clinical management of early-stage NSCLC

In round 1, consensus on clinical management of early-stage NSCLC was achieved in 9 of the 15 statements (60%). The expert panel unanimously agreed that patients with stage I and II NSCLC undergo surgery in routine clinical practice (statements 2a–2c). However, the panelists expressed a lack of consensus regarding surgical management of stage IIIA NSCLC (statement 2d). This item was not reassessed in round 2 as it reflected local/routine clinical practice, and the panel response was not expected to change. A unanimous consensus was reached regarding the use of chemotherapy in patients with stage II (90.6%) and IIIA (100.0%) (statements 5b and 5c) according to local clinical practice. Consensus was not achieved on the use of adjuvant chemotherapy in stage IB NSCLC; however, this statement was not evaluated in round 2 as the panel response mirrored the routine clinical practice. Regarding biomarker testing after surgical resection, 84.4% of the panelists agreed that *EGFR* mutation testing should be done after surgery (statement 6a), although consensus was not achieved on the appropriateness of testing *ALK* (statement 6b), *ROS1* (statement 6c), and PD-L1 expression (statement 6d). These 3 non-consensus statements in round 1 also failed to reach consensus in round 2. Consensus was achieved regarding whether follow-up has to be done by the medical oncology service regardless of the use of adjuvant therapy after surgery (68.8%) (statement 7) (Table 1).

**Table 1:** Results of the two-step Delphi process for the statements on clinical management of early-stage NSCLC

Statements	Round	Rejected (scores 1–3)	Undetermined (scores 4–6)	Accepted (scores 7–9)	Consensus
		*N* (%)	*N* (%)	*N* (%)	
1. The percentage of NSCLC patients with resectable disease at diagnosis is 25–30%	1	4 (12.5)	5 (15.6)	23 (71.9)	Consensus
2. Surgical candidates with NSCLC who undergo surgery have:					
2a. Stage IA	1	0 (0.0)	0 (0.0)	32 (100.0)	Consensus
2b. Stage IB	1	0 (0.0)	0 (0.0)	32 (100.0)	Consensus
2c. Stage II	1	0 (0.0)	0 (0.0)	32 (100.0)	Consensus
2d. Stage IIIA	1	3 (9.4)	19 (59.4)	10 (31.3)	No consensus^a^
3. All patients who have completed surgical resection are referred to the Medical Oncology Service	1	2 (6.3)	9 (28.1)	21 (65.6)	Consensus^b^
4. Only patients who are candidates to adjuvant chemotherapy are referred to Medical Oncology Service after complete surgical resection	1	17 (53.1)	2 (6.3)	13 (40.6)	No consensus^a^
5. Adjuvant chemotherapy is an adequate treatment strategy in patients with:					
5a. Stage IB	1	9 (28.1)	12 (37.5)	11 (34.4)	No consensus^a^
5b. Stage II	1	0 (0.0)	3 (9.4)	29 (90.6)	Consensus
5c. Stage IIIA	1	0 (0.0)	0 (0.0)	32 (100.0)	Consensus
6. After surgery, the following biomarkers should be tested in the surgical specimen					
6a. EGFR	1	2 (6.3)	3 (9.4)	27 (84.4)	Consensus
6b. ALK	1	9 (28.1)	8 (25.0)	15 (46.9)	No consensus
	2	9 (29.0)	9 (29.0)	13 (41.9)	No consensus
6c. ROS1	1	11 (34.4)	10 (31.3)	11 (34.4)	No consensus
	2	10 (32.3)	8 (25.8)	10 (32.3)	No consensus
6d. PD-L1	1	11 (34.4)	7 (21.9)	14 (43.8)	No consensus
	2	4 (12.9)	8 (25.8)	19 (61.3)	No consensus
7. NSCLC Patients are followed in the Medical Oncology Service regardless of receipt of adjuvant chemotherapy after surgery	1	2 (6.3)	8 (25.0)	22 (68.8)	Consensus

*EGFR* epidermal growth factor receptor, *NSCLC* non-small cell lung cancer

^a^This statement was not reassessed in round 2 as it reflected local/routine clinical practice, and the panel response was not expected to change according to scientific committee criteria

^b^This statement was considered to have achieved consensus as it almost achieved the 66.6% threshold required for consensus

### Role of adjuvant therapy in early-stage NSCLC

Consensus on the role of adjuvant therapy in early-stage NSCLC was reached in 7 of the 9 statements (77.8%) evaluated in round 1. There was no consensus on whether adjuvant chemotherapy adversely impact patients´ quality of life (statement 5). Moderate agreement (65.6%) was reached among panelists on whether DFS prolongation is enough to consider that a specific therapy is effective in the adjuvant setting (statement 3). Additionally, panelists expressed a lack of consensus on the potential correlation of DFS and OS in the adjuvant scenario (statement 7). None of the two non-consensus statements evaluated in round 2 (statements 5 and 7) reached an agreement. Nearly 97% agreed that OS is the most relevant goal of adjuvant therapy (statement 6). A strong consensus was reached regarding the adverse impact of disease relapse on patients´ quality of life (96.9%) and employment status (100.0%) statements 8 and 9) (Table 2).

**Table 2:** Results of the two-step Delphi process for the statements on the role of adjuvant therapy in early-stage NSCLC

Statements	Round	Rejected (scores 1–3)	Undetermined (scores 4–6)	Accepted (scores 7–9)	Consensus
		*N* (%)	*N* (%)	*N* (%)	
1. Despite treatment with surgery with or without adjuvant chemotherapy, the risk of relapse is high	1	0 (0.0)	4 (12.5)	28 (87.5)	Consensus
2. The impact of adjuvant therapy on survival is limited	1	0 (0.0)	4 (12.5)	28 (87.5)	Consensus
3. Adjuvant therapy is considered to be effective if it enables disease-free survival prolongation	1	6 (18.8)	5 (15.6)	21 (65.6)	Consensus^a^
4. The potential toxicity of adjuvant chemotherapy impacts on treatment decision regarding its administration	1	0 (0.0)	5 (15.6)	27 (84.4)	Consensus
5. Adjuvant chemotherapy adversely impact patient´s quality of life	1	9 (28.1)	13 (40.6)	10 (31.3)	No consensus
	2	12 (38.7)	10 (32.3)	9 (29.0)	No consensus
6. Improvement of overall survival is the most relevant goal of adjuvant therapy	1	0 (0.0)	1 (3.1)	31 (96.9)	Consensus
7. Disease-free survival correlates with overall survival in the adjuvant setting	1	3 (9.4)	10 (31.3)	19 (59.4)	No consensus
	2	2 (6.5)	10 (32.3)	19 (61.3)	No consensus
8. Disease relapse adversely impacts patient´s quality of life	1	0 (0.0)	1 (3.1)	31 (96.9)	Consensus
9. Disease relapse negatively impacts patient´s employment status	1	0 (0.0)	0 (0.0)	32 (100.0)	Consensus

^a^This statement was considered to have achieved consensus as the voting percentage was nearly the 66.6% threshold for consensus

### Role of adjuvant therapy in early-stage NSCLC with sensitizing EGFR mutation

In round 1, panel experts agreed with 8 of the 12 statements (66.7%) regarding the role of adjuvant therapy in early-stage NSCLC with sensitizing EGFR mutation. Consensus was not achieved on the inconvenience of prolonged duration of osimertinib for the patient (item 7). Additionally, there was a lack of consensus regarding whether the implementation of *EGFR* mutation testing in resected early-stage NSCLC could limit the use of adjuvant osimertinib (statement 9). Consensus was neither reached on the utility of minimal residual disease (MRD) detection as an alternative option for early detection of relapse (statement 10). Lastly, there was no consensus regarding whether the use of adjuvant osimertinib may limit therapeutic options upon recurrence on osimertinib (statement 12). Two statements were reformulated to clarify their contents (statements 9 and 12). Of note, a high degree of consensus (90.6%) was achieved on the clinically significant DFS benefit of osimertinib observed in the ADAURA study (statement 3), and on the fact that osimertinib could shift the treatment paradigm in patients with stage IB–IIIA *EGFR*-mutated NSCLC (71.9%) (statement 4). Finally, four statements that did not achieve consensus or were reformulated were evaluated in round 2, and none of them met consensus (statements 7, 9, 10, and 12) (Table 3).

**Table 3:** Results of the two-step Delphi process for the statements relating to the role of adjuvant therapy in early-stage NSCLC with EGFR sensitizing mutation

Statements	Round	Rejected (scores 1–3)	Undetermined (scores 4–6)	Accepted (scores 7–9)	Consensus
1. There is a rationale for using osimertinib as adjuvant therapy for advanced NSCLC with sensitizing EGFR mutation based on consistency of clinically significant results	1	1 (3.1)	3 (9.4)	28 (87.5)	Consensus
2. There is a rationale for using osimertinib as adjuvant treatment for NSCLC with EGFR sensitizing mutation based on clinical evidence demonstrating the CNS activity	1	1 (3.1)	4 (12.5)	27 (84.4)	Consensus
3. Based on the data of the ADAURA study interim analysis, the DFS benefit of osimertinib is clinically significant	1	1 (3.1)	2 (6.3)	29 (90.6)	Consensus
4. Based on the data of the ADAURA study interim analysis, osimertinib will change the treatment paradigm in patients with stage IB–IIIA EGFR-mutated NSCLC	1	2 (6.3)	7 (21.9)	23 (71.9)	Consensus
5. The magnitude of the DFS benefit of osimertinib is enough for its therapeutic indication in the adjuvant setting	1	4 (12.5)	7 (21.9)	21 (65.6)	Consensus^a^
6.The DFS benefit of osimertinib across all subgroups in the ADAURA study is of relevance	1	1 (3.1)	6 (18.8)	25 (78.1)	Consensus
7. The prolonged duration of osimertinib treatment may be inconvenient for the patient	1	6 (18.8)	6 (18.8)	20 (62.5)	No consensus
	2	10 (32.3)	10 (32.3)	11 (35.5)	No consensus
8. The budget impact of osimertinib is notable	1	0 (0.0)	5 (15.6)	27 (84.4)	Consensus
9. The requirement of EGFR mutation testing in patients with resected disease involves a limitation to the incorporation of osimertinib as an adjuvant therapy	1	6 (18.8)	6 (18.8)	20 (62.5)	No consensus^b^
	2	17 (54.8)	7 (22.6)	7 (22.6)	No consensus
10. Alternative options for early detection of relapse, such as minimal residual disease detection, should be considered	1	2 (6.3)	10 (31.3)	20 (62.5)	No consensus
	2	3 (9.7)	8 (25.8)	20 (64.5)	No consensus
11. Adjuvant chemotherapy treatment cannot be dispensed with now when it is indicated in early-stage NSCLC patients if adjuvant osimertinib is administered	1	6 (18.8)	4 (12.5)	22 (68.8)	Consensus
12. Osimertinib administration as an adjuvant therapy may limit therapeutic options in case of recurrence	1	14 (43.8)	11 (34.4)	7 (21.9)	No consensus^c^
	2	13 (41.9)	8 (25.8)	10 (32.3)	No consensus

*CNS* central nervous system, *EGFR* epidermal growth factor receptor, *NSCLC* non-small cell lung cancer

^a^This statement was considered to have achieved consensus as the voting percentage was nearly the 66.6% threshold for consensus

^b^This statement was modified to be evaluated in round 2. The initial proposal of this statement was as follows: “The incorporation of osimertinib in the adjuvant setting require EGFR mutation testing, which may result in a limitation”

^c^This statement was modified to be evaluated in round 2. The initial proposal of this statement was as follows: “Limitation of the therapeutic options in case of recurrence determines the administration of osimertinib as adjuvant treatment”

## Discussion

This Delphi panel study was able to reach consensus among medical oncologists in relevant aspects of early-stage NSCLC management, particularly focusing on the role and use of adjuvant therapy in such scenario. Nevertheless, this Delphi process also revealed uncertain issues, particularly related to molecular testing in the adjuvant setting.

This Delphi panel study reveals that surgery remains the standard of care for stage I and II NSCLC. As expected, there was no consensus regarding whether patients with stage IIIA NSCLC are surgical candidates in a real-world setting, probably since stage III NSCLC is a highly heterogeneous disease requiring personalized multimodal treatment [22]. Adjuvant chemotherapy may improve survival, and accordingly, the panel unanimously supported the use of adjuvant chemotherapy after complete resection in patients with stage IIIA NSCLC. A 90% consensus was also achieved regarding the use of adjuvant chemotherapy in stage II NSCLC, based on the updated European Society for Medical Oncology (ESMO) guideline recommendations (grade IA) [1]. However, consensus was not reached among panelists on its use in stage IB NSCLC patients, in whom the risk of relapse is lower. Nevertheless, nearly 35% of panelists supported its use also in this situation.

Consensus was reached regarding referral to medical oncology following surgical resection. Approximately 69% of panelists stated that NSCLC patients are referred to the medical oncology service to decide about adjuvant systemic therapy after debating the pros and cons with the patient.

The survival benefit of adjuvant chemotherapy in patients with resected NSCLC remains poor [5, 23–25]. Moderate consensus agreement (65.6% agreed) was achieved on whether an adjuvant therapy should be considered an effective approach as long as it improves DFS. Indeed, a unanimous consensus was reached on the relevance of OS improvement as the most important goal of adjuvant therapy (97%). The uncertainty among experts regarding the potential correlation between DFS and OS may in part be due to the lack of evidence demonstrating translation of DFS improvement into OS benefit with targeted therapies such as gefitinib and erlotinib in the adjuvant setting for *EGFR*-mutant NSCLC [26–28].

In this scenario, efforts have been made to explore new treatment strategies that could improve outcomes for patients with early-stage NSCLC. The third-generation *EGFR* TKI osimertinib has demonstrated a clear DFS benefit for early-stage lung cancer patients with *EGFR* mutations [8]. Accordingly, panelists acknowledged the significant and clinically meaningful DFS benefit of osimertinib observed in the ADAURA study. Panel experts also highlighted the relevance of the DFS benefit of osimertinib across all subgroups in the ADAURA study, including disease stages IB–IIIA, race, use of adjuvant chemotherapy, and *EGFR* common mutation subtype.

In early-stage NSCLC, overall survival improvement along with changing the natural history of the disease are relevant endpoints. Osimertinib has showed a significant reduction in the rate of central nervous system (CNS) relapses (1%) compared with placebo (10%) [15]. These findings are particularly noteworthy since CNS is a common site of relapse, typically associated with poor prognosis and limited therapeutic strategies available in *EGFR*-mutated NSCLC. Accordingly, the Delphi experts widely concurred that there was a rationale for adjuvant osimertinib use in *EGFR*-mutated NSCLC based on the clinically meaningful CNS activity observed with adjuvant osimertinib.

Nearly 66% of panelists concurred that the magnitude of DFS benefit with osimertinib is enough for its indication as adjuvant therapy for resected early-stage NSCLC. The uncertainty (22%) or disagreement (12%) among experts regarding this issue might be related to the immaturity of OS data in the ADAURA study. Nevertheless, panel experts agreed that adjuvant osimertinib could change the treatment paradigm in completely resected stage IB–IIIA NSCLC (72%).

In addition to the lack of mature OS data, the high cost and the 3-year treatment duration of osimertinib may hamper its widespread use. Consensus was not achieved on the inconvenience of prolonged duration of osimertinib treatment. Overall, 32% of panelists did not agree that the 3-year treatment period may be troublesome for the patient. As the panelists recognized (84% agreed), the budget impact of osimertinib is notable. However, although a cost-effectiveness analysis has not been performed yet, effective relapse prevention may avoid the need of additional expensive further treatment for recurrent disease.

The use of osimertinib in the adjuvant setting requires molecular testing for all patients with resected *EGFR*-mutated NSCLC. Nevertheless, panel experts did not perceive genomic profiling as a potential limitation for the use of adjuvant osimertinib, and most panelists (84%) agreed that *EGFR* mutation testing should be done after surgical resection according to guideline recommendations [29]. However, consensus was not achieved on the recommendation for testing other genomic alterations or PD-L1 expression probably due to the lack of specific targeted therapies and immunotherapy results that were available when the Delphi survey was performed.

Although detection of circulating tumor DNA (ctDNA) and MRD by liquid biopsy after curative-intent surgery may be a prognostic and dynamic marker of early recurrence, there was no consensus about the role of MRD detection as an alternative option for early detection of relapse. Indeed, several studies are exploring the potential value of ctDNA monitoring to detect the MRD in resected early-stage NSCLC, based on small available evidence [30–32].

No consensus was reached on the potential limitation of therapeutic options available upon recurrence on osimertinib, although nearly 42% of panelists did not perceive this issue as a limitation to its use in the adjuvant setting. In this context, patients who develop disease recurrence despite treatment with adjuvant osimertinib should probably undergo tumor or liquid biopsy to assess for acquired resistance mechanisms. Nevertheless, further research is required to delineate more clearly the optimal treatments upon recurrence on osimertinib.

Data from Delphi studies focused on NSCLC are still limited and have mostly focused on other aspects of disease management. A Delphi panel has previously described treatment patterns, use of resources and costs associated with the treatment of metastatic NSCLC in Spain [33]. Other Delphi panel studies have been conducted to establish recommendations on surgical decision-making in NSCLC patients (34) or NSCLC surveillance after stereotactic ablative radiation therapy [35]. Recently, a Delphi study was conducted for optimized treatment strategies for patients with advanced NSCLC with EGFR sensitizing mutations in Spain [36]. However, to our knowledge, the study presented here is the first Delphi consensus focused on the clinical management of early-stage EGFR-mutated NSCLC, which makes it particularly interesting after the publication of the ADAURA study results.

This expert panel study has several limitations. First, the results should be interpreted in the context of a national Delphi project and panelists’ responses reflect the oncology practice in Spain. Second, an arbitrary consensus threshold of 66% was set as a frequent consensus cut-off, and we should consider that a different threshold would yield different findings. Lastly, panel experts did not have the opportunity to re-evaluate the non-consensus statements considering other experts’ feedback as comments from panelists were not collected. The strengths of this study include the high participation of the expert panelists in the 2-round Delphi process (97%) from hospitals distributed throughout the country, ensuring geographical representativeness of different Spanish regions.

In conclusion, this Delphi study provides valuable insights into relevant questions in the management of patients with early-stage NSCLC. The expert consensus emphasizes the role of adjuvant treatment and support the use of osimertinib in the adjuvant scenario. Additionally, this Delphi process also identified specific issues that remain unresolved in the setting of early-stage NSCLC. Efforts should be therefore focused on those questions for which panel experts expressed uncertainty, including the impact on OS, the duration of adjuvant therapy with osimertinib, the role and implications of molecular testing in the adjuvant scenario, and the financial impact of the whole process.

## Supplementary Information

Below is the link to the electronic supplementary material.

Supplementary file1 (DOCX 40 KB)

Supplementary file2 (EPS 2510 KB)
