# Supplementary material for: A Delphi consensus panel about clinical management of early-stage EGFR-mutated non-small cell lung cancer (NSCLC) in Spain: a Delphi consensus panel study
Source: Clin Transl Oncol. 2022 Sep 27;25(1):283–91. doi: 10.1007/s12094-022-02941-5 (PMC9813031; doi:10.1007/s12094-022-02941-5)
Supplement: Supplementary file 1 — Supplementary file1 (DOCX 40 KB) [file 12094_2022_2941_MOESM1_ESM.docx]

**Supplementary Table 1.** **Results of the two-step Delphi process for the statements on clinical management of early-stage NSCLC**

| **Statements** | **Round** | **1** | **2** | **3** | **4** | **5** | **6** | **7** | **8** | **9** | **Consensus** |
| --- | --- | --- | --- | --- | --- | --- | --- | --- | --- | --- | --- |
|  |  | **N (%)** | **N (%)** | **N (%)** | **N (%)** | **N (%)** | **N (%)** | **N (%)** | **N (%)** | **N (%)** |  |
| 1. The percentage of NSCLC patients with resectable disease at diagnosis is 25-30% | 1 | 1 (3.1) | 0 (0.0) | 3 (9.4) | 2 (6.3) | 1 (3.1) | 2 (6.3) | 9 (28.1) | 5 (15.6) | 9 (28.1) | Consensus |
| 2. Surgical candidates with NSCLC who undergo surgery have: |  |  |  |  |  |  |  |  |  |  |  |
| 2a. Stage IA | 1 | 0 (0.0) | 0 (0.0) | 0 (0.0) | 0 (0.0) | 0 (0.0) | 0 (0.0) | 0 (0.0) | 7 (21.9) | 25 (78.1) | Consensus |
| 2b. Stage IB | 1 | 0 (0.0) | 0 (0.0) | 0 (0.0) | 0 (0.0) | 0 (0.0) | 0 (0.0) | 0 (0.0) | 8 (18.8) | 26 (81.3) | Consensus |
| 2c. Stage II | 1 | 0 (0.0) | 0 (0.0) | 0 (0.0) | 0 (0.0) | 0 (0.0) | 0 (0.0) | 3 (9.4) | 8 (25.0) | 21 (65.6) | Consensus |
| 2d. Stage IIIA | 1 | 1 (3.1) | 1 (3.1) | 1 (3.1) | 1 (3.1) | 4 (12.5) | 14 (43.8) | 7 (21.9) | 0 (0.0) | 3 (9.4) | No consensus^(1)^ |
| 3. All patients who have completed surgical resection are referred to the Medical Oncology Service | 1 | 1 (3.1) | 0 (0.0) | 1 (3.1) | 3 (9.4) | 3 (9.4) | 3 (9.4) | 2 (6.3) | 8 (25.0) | 11 (34.4) | Consensus^(2)^ |
| 4. Only patients who are candidates to adjuvant chemotherapy are referred to Medical Oncology Service after complete surgical resection | 1 | 10 (31.3) | 4 (12.5) | 3 (9.4) | 1 (3.1) | 1 (3.1) | 0 (0.0) | 2 (6.3) | 5 (15.6) | 6 (18.8) | No consensus^(1)^ |
| 5. Adjuvant chemotherapy is an adequate treatment strategy in patients with: |  |  |  |  |  |  |  |  |  |  |  |
| 5a. Stage IB | 1 | 3 (9.4) | 4 (12.5) | 2 (6.3) | 2 (6.3) | 5 (15.6) | 5 (15.6) | 5 (15.6) | 3 (9.4) | 3 (9.4) | No consensus^(1)^ |
| 5b. Stage II | 1 | 0 (0.0) | 0 (0.0) | 0 (0.0) | 2 (6.3) | 1 (3.1) | 0 (0.0) | 3 (9.4) | 3 (9.4) | 23 (71.9) | Consensus |
| 5c. Stage IIIA | 1 | 0 (0.0) | 0 (0.0) | 0 (0.0) | 0 (0.0) | 0 (0.0) | 0 (0.0) | 1 (3.1) | 4 (12.5) | 27 (84.4) | Consensus |
| 6. After surgery, the following biomarkers should be tested in the surgical specimen |  |  |  |  |  |  |  |  |  |  |  |
| 6a. EGFR | 1 | 2 (6.3) | 0 (0.0) | 0 (0.0) | 1 (3.1) | 1 (3.1) | 1 (3.1) | 5 (15.6) | 8 (25.0) | 14 (43.8) | Consensus |
| 6b. ALK | 1  2 | 4 (12.5)  3 (9.7) | 3 (9.4)  2 (6.5) | 2 (6.3)  4 (12.9) | 2 (6.3)  1 (3.2) | 4 (12.5)  7 (22.6) | 2 (6.3)  1 (3.2) | 4 (12.5)  4 (12.9) | 2 (6.3)  2 (6.5) | 9 (28.1)  7 (22.6) | No consensus  No consensus |
| 6c. ROS1 | 1  2 | 6 (18.8)  5 (16.1) | 4 (12.5)  2 (6.5) | 1 (3.1)  6 (19.4) | 3 (9.4)  2 (6.5) | 5 (15.6)  5 (16.1) | 2 (6.3)  1 (3.2) | 2 (6.3)  3 (9.7) | 2 (6.3)  1 (3.2) | 7 (21.9)  6 (19.4) | No consensus  No consensus |
| 6d. PDL1 | 1  2 | 5 (15.6)  2 (6.5) | 3 (9.4)  1 (3.2) | 3 (9.4)  1 (3.2) | 2 (6.3)  1 (3.2) | 3 (9.4)  4 (12.9) | 2 (6.3)  3 (9.7) | 3 (9.4)  6 (19.4) | 1 (3.1)  2 (6.5) | 10 (31.3)  11 (35.5) | No consensus  No consensus |
| 7. NSCLC Patients are followed in the Medical Oncology Service regardless of receipt of adjuvant chemotherapy after surgery | 1 | 1 (3.1) | 1 (3.1) | 0 (0.0) | 0 (0.0) | 5 (15.6) | 3 (9.4) | 6 (18.8) | 3 (9.4) | 13 (40.6) | Consensus |
| EGFR: epidermal growth factor receptor; NSCLC: non-small cell lung cancer  ^(1)^ This statement was not reassessed in round 2 as it reflected local/routine clinical practice, and the panel response was not expected to change according to scientific committee criteria. ^(2)^ This statement was considered to have achieved consensus as it almost achieved the 66.6% threshold required for consensus. | | | | | | | | | | | |

**Supplementary Table 2. Results of the two-step Delphi process for the statements on the role of adjuvant therapy in early-stage NSCLC**

| **Statements** | **Round** | **1** | **2** | **3** | **4** | **5** | **6** | **7** | **8** | **9** | **Consensus** |
| --- | --- | --- | --- | --- | --- | --- | --- | --- | --- | --- | --- |
|  |  | **N (%)** | **N (%)** | **N (%)** | **N (%)** | **N (%)** | **N (%)** | **N (%)** | **N (%)** | **N (%)** |  |
| 1. Despite treatment with surgery with or without adjuvant chemotherapy, the risk of relapse is high | 1 | 0 (0.0) | 0 (0.0) | 0 (0.0) | 0 (0.0) | 2 (6.3) | 2 (6.3) | 9 (28.1) | 6 (18.8) | 13 (40.6) | Consensus |
| 2. The impact of adjuvant therapy on survival is limited | 1 | 0 (0.0) | 0 (0.0) | 0 (0.0) | 0 (0.0) | 2 (6.3) | 2 (6.3) | 4 (12.5) | 8 (25.0) | 16 (50.0) | Consensus |
| 3. Adjuvant therapy is considered to be effective if it enables disease-free survival prolongation | 1 | 1 (3.1) | 0 (0.0) | 5 (15.6) | 0 (0.0) | 3 (9.4) | 2 (6.3) | 5 (15.6) | 7 (21.9) | 9 (28.1) | Consensus^(1)^ |
| 4. The potential toxicity of adjuvant chemotherapy impacts on treatment decision regarding its administration | 1 | 0 (0.0) | 0 (0.0) | 0 (0.0) | 1 (3.1) | 3 (9.4) | 1 (3.1) | 9 (28.1) | 9 (28.1) | 9 (28.1) | Consensus |
| 5. Adjuvant chemotherapy adversely impact patient´s quality of life | 1  2 | 1 (3.1)  1 (3.2) | 1 (3.1)  3 (9.7) | 7 (21.9)  8 (25.8) | 2 (6.3)  1 (3.2) | 6 (18.8)  3 (9.7) | 5 (15.6)  6 (19.4) | 5 (15.6)  7 (22.6) | 3 (9.4)  1 (3.2) | 2 (6.3)  1 (3.2) | No consensus  No consensus |
| 6. Improvement of overall survival is the most relevant goal of adjuvant therapy | 1 | 0 (0.0) | 0 (0.0) | 0 (0.0) | 0 (0.0) | 0 (0.0) | 1 (3.1) | 2 (6.3) | 8 (25.0) | 21 (65.6) | Consensus |
| 7. Disease-free survival correlates with overall survival in the adjuvant setting | 1  2 | 2 (6.3)  1 (3.2) | 1 (3.1)  0 (0.0) | 0 (0.0)  1 (3.2) | 0 (0.0)  1 (3.2) | 6 (18.8)  4 (12.9) | 4 (12.5)  5 (16.1) | 9 (28.1)  12 (38.7) | 9 (28.1)  6 (19.4) | 1 (3.1)  1 (3.2) | No consensus  No consensus |
| 8. Disease relapse adversely impacts patient´s quality of life | 1 | 0 (0.0) | 0 (0.0) | 0 (0.0) | 0 (0.0) | 1 (3.1) | 0 (0.0) | 1 (3.1) | 7 (21.9) | 23 (71.9) | Consensus |
| 9. Disease relapse negatively impacts patient´s employment status | 1 | 0 (0.0) | 0 (0.0) | 0 (0.0) | 0 (0.0) | 0 (0.0) | 0 (0.0) | 3 (9.4) | 8 (25.0) | 21 (65.6) | Consensus |
| ^(1)^ This statement was considered to have achieved consensus as the voting percentage was nearly the 66.6% threshold for consensus | | | | | | | | | | | |

**Supplementary Table 3. Results of the two-step Delphi process for the statements relating to the role of adjuvant therapy in early-stage NSCLC with EGFR sensitizing mutation**

| **Statements** | **Round** | **1** | **2** | **3** | **4** | **5** | **6** | **7** | **8** | **9** | **Consensus** |
| --- | --- | --- | --- | --- | --- | --- | --- | --- | --- | --- | --- |
|  |  | **N (%)** | **N (%)** | **N (%)** | **N (%)** | **N (%)** | **N (%)** | **N (%)** | **N (%)** | **N (%)** |  |
| 1. There is a rationale for using osimertinib as adjuvant therapy for advanced NSCLC with sensitizing EGFR mutation based on consistency of clinically significant results. | 1 | 0 (0.0) | 0 (0.0) | 1 (3.1) | 0 (0.0) | 1 (3.1) | 2 (6.3) | 7 (21.9) | 8 (25.0) | 13 (40.6) | Consensus |
| 2. There is a rationale for using osimertinib as adjuvant treatment for NSCLC with EGFR sensitizing mutation based on clinical evidence demonstrating the CNS activity. | 1 | 0 (0.0) | 0 (0.0) | 1 (3.1) | 0 (0.0) | 2 (6.3) | 2 (6.3) | 5 (15.6) | 8 (25.0) | 14 (43.8) | Consensus |
| 3. Based on the data of the ADAURA study interim analysis, the DFS benefit of osimertinib is clinically significant | 1 | 1 (3.1) | 0 (0.0) | 0 (0.0) | 0 (0.0) | 1 (3.1) | 1 (3.1) | 5 (15.6) | 8 (25.0) | 16 (50.0) | Consensus |
| 4. Based on the data of the ADAURA study interim analysis, osimertinib will change the treatment paradigm in patients with stage IB-IIIA EGFR-mutated NSCLC | 1 | 1 (3.1) | 0 (0.0) | 1 (3.1) | 0 (0.0) | 3 (9.4) | 4 (12.5) | 7 (21.9) | 5 (15.6) | 11 (34.4) | Consensus |
| 5. The magnitude of the DFS benefit of osimertinib is enough for its therapeutic indication in the adjuvant setting | 1 | 1 (3.1) | 0 (0.0) | 3 (9.4) | 0 (0.0) | 4 (12.5) | 3 (9.4) | 8 (25.0) | 4 (12.5) | 9 (28.1) | Consensus^(1)^ |
| 6.The DFS benefit of osimertinib across all subgroups in the ADAURA study is of relevance | 1 | 1 (3.1) | 0 (0.0) | 0 (0.0) | 3 (9.4) | 1 (3.1) | 2 (6.3) | 7 (21.9) | 5 (15.6) | 13 (40.6) | Consensus |
| 7. The prolonged duration of osimertinib treatment may be inconvenient for the patient | 1  2 | 3 (9.4)  4 (12.9) | 1 (3.1)  2 (6.5) | 2 (6.3)  4 (12.9) | 1 (3.1)  3 (9.7) | 2 (6.3)  3 (9.7) | 3 (9.4)  4 (12.9) | 12 (37.5)  6 (19.4) | 5 (15.6)  4 (12.9) | 3 (9.4)  1 (3.2) | No consensus  No consensus |
| 8. The budget impact of osimertinib is notable | 1 | 0 (0.0) | 0 (0.0) | 0 (0.0) | 1 (3.1) | 2 (6.3) | 2 (6.3) | 3 (9.4) | 9 (28.1) | 15 (46.9) | Consensus |
| 9. The requirement of EGFR mutation testing in patients with resected disease involves a limitation to the incorporation of osimertinib as an adjuvant therapy | 1  2 | 2 (6.3)  8 (25.8) | 3 (9.4)  7 (22.6) | 1 (3.1)  2 (6.5) | 1 (3.1)  1 (3.2) | 2 (6.3)  1 (3.2) | 3 (9.4)  5 (16.1) | 6 (18.8)  2 (6.5) | 5 (15.6)  4 (12.9) | 9 (28.1)  1 (3.2) | No consensus^(2)^  No consensus |
| 10. Alternative options for early detection of relapse, such as minimal residual disease detection, should be considered | 1  2 | 1 (3.1)  0 (0.0) | 0 (0.0)  0 (0.0) | 1 (3.1)  3 (9.7) | 3 (9.4)  2 (6.5) | 3 (9.4)  5 (16.1) | 4 (12.5)  1 (3.2) | 2 (6.3)  9 (29.0) | 7 (21.9)  4 (12.9) | 11 (34.4)  7 (22.6) | No consensus  No consensus |
| 11. Adjuvant chemotherapy treatment cannot be dispensed with now when it is indicated in early-stage NSCLC patients if adjuvant osimertinib is administered | 1 | 1 (3.1) | 2 (6.3) | 3 (9.4) | 0 (0.0) | 2 (6.3) | 2 (6.3) | 6 (18.8) | 6 (18.8) | 10 (31.3) | Consensus |
| 12. Osimertinib administration as an adjuvant therapy may limit therapeutic options in case of recurrence | 1  2 | 8 (25.0)  5 (16.1) | 3 (9.4)  2 (6.5) | 3 (9.4)  6 (19.4) | 3 (9.4)  2 (6.5) | 6 (18.8)  2 (6.5) | 2 (6.3)  4 (12.9) | 5 (15.6)  6 (19.4) | 1 (3.1)  2 (6.5) | 1 (3.1)  2 (6.5) | No consensus^(3)^  No consensus |
| CNS: central nervous system; EGFR: epidermal growth factor receptor; NSCLC: non-small cell lung cancer. ^(1)^ This statement was considered to have achieved consensus as the voting percentage was nearly the 66.6% threshold for consensus; ^(2)^ This statement was modified to be evaluated in round 2. The initial proposal of this statement was as follows: “The incorporation of osimertinib in the adjuvant setting require EGFR mutation testing, which may result in a limitation”; ^(3)^ This statement was modified to be evaluated in round 2. The initial proposal of this statement was as follows: “Limitation of the therapeutic options in case of recurrence determines the administration of osimertinib as adjuvant treatment” | | | | | | | | | | | |
